# Cyclodextrin Rotaxanes of Pt Complexes and Their Conversion to Pt Nanoparticles

**DOI:** 10.3390/molecules25235617

**Published:** 2020-11-29

**Authors:** Yuji Suzaki, Yuhei Fujii, Kohtaro Osakada

**Affiliations:** 1 Laboratory for Chemistry and Life Science, Tokyo Institute of Technology, 4259 Nagatsuta, Yokohama 226-8503, Japan; ysuzaki@gmail.com (Y.S.); funky.southern.ymt@gmail.com (Y.F.); 2 National Institute of Advanced Industrial Science and Technology (AIST), Tsukuba Central 5, 1-1-1 Higashi, Tsukuba, Ibaraki 305-8565, Japan

**Keywords:** platinum, cyclodextrin, rotaxane, nanoparticles

## Abstract

The cationic Pt complex (Pt(NC_6_H_4_-C_6_H_4_N-(CH_2_)_10_-O(C_6_H_3_-3,5-(OMe)_2_)(MeN-(CH_2_CH_2_NMe_2_)_2_))^+^ was prepared by the reaction of alkylbipyridinium ligand with a nitrateplatinum(II) complex. Mixing the complex and α- and β-cyclodextrins in aqueous media produced the corresponding [2]rotaxanes with 1:1 stoichiometry. γ-Cyclodextrin and the Pt complex formed a rotaxane having components in a 1:1 or 2:1 molar ratio. The results of mass and nuclear magnetic resonance (NMR) measurements confirmed the rotaxane structures of the Pt complexes. Transmission electron microscopy (TEM) and atomic force microscope (AFM) analyses revealed the formation of micelles or vesicles. The addition of NaBH_4_ to the rotaxanes in aqueous media formed Pt nanoparticles with diameters of 1.3–2.8 nm, as characterized by TEM. The aggregated size of the nanoparticles formed from the rotaxane did not change even at 70 °C, and they showed higher thermal stability than those obtained from the reduction of the cyclodextrin-free Pt complex.

## 1. Introduction

Pt nanoparticles have been investigated because of their usability in catalysis, fuel cell materials, and sensing materials [1]. A common preparation procedure of Pt nanoparticles involves the preparation of precursors from H_2_PtCl_6_ and template compounds, such as surfactants and polymers dispersed in aqueous media. The subsequent reduction of the Pt(IV) species results in the deposition of the Pt nanoparticles under mild conditions. Surfactants have a dual role in the process: coordination with the Pt-containing precursors to keep their colloidal aggregation in the aqueous media, and protection of the formed nanoparticles by preventing their further growth. A typical preparation procedure was reported as follows. The addition of an aqueous solution of H_2_PtCl_6_ to an isooctane solution of sodium bis(2-ethylhexyl) sulfosuccinate (AOT) formed a colloidal suspension containing Pt salt. An NaBH_4_ reduction of the precursor suspension at 4 °C yielded Pt nanoparticles with an average diameter of 13 nm [2]. Tetradecyltrimethylammonium bromide (TTAB) and sodium citrate were also used as templates for the synthesis of the Pt nanoparticles [3,4]. Polymers such as polyvinylpyrrolidone (PVP) [5,6] and poly(acrylic acid) (PA) [7] function as templates to stabilize metal nanoparticles effectively. Natural polymers, such as silk, were reported to function as the template in the Pt nanoparticle formation [8]. The reduction of the Pt(IV) precursors with diol and alcohol at elevated temperatures also produced the Pt nanoparticles [9]. Typically, Toste et al. heated an ethylene glycol solution of NaOH and H_2_PtCl_6_ at 160 °C, and neutralized the mixture by using HCl [10]. Dispersing the resulting mixture in an ethanol solution of PVP caused the formation of Pt nanoparticles with a diameter of 1.5 nm. Conversely, the sequential addition of the aqueous H_2_PtCl_6_ solution and PVP to methanol, followed by heating the mixture, caused the formation of Pt particles with a diameter of 2.9 nm. The Pt particles supported by silica were used as the catalyst for the selective synthesis of heterocyclic compounds.

Rotaxanes are molecules consisting of more than one component, i.e., macrocyclic and linear compounds, with the former encircling the latter. Major research interest in rotaxanes pertains to their applicability as molecular machines, molecular devices [11,12,13,14,15,16,17,18,19,20], and supramolecular catalysts [21,22,23,24,25]. Cyclodextrins have tube-like molecular structures and form rotaxanes with linear-shaped organic and polymer molecules. Cyclodextrins and transition-metal complexes having alkyl or alkylene ligands have been investigated for several decades [26,27,28,29,30]. Studies on the cyclodextrin rotaxanes of hydrophobic organic polymers have revealed their aggregation in the solid state caused by the intermolecular interaction of cyclodextrin components [31,32,33,34,35,36,37,38,39,40]. Previously, we prepared pseudo-rotaxanes composed of 4-alkyl-4,4′-bipyridinium, with a bulky 3,5-dimethylphenyl end (linear component) and cyclodextrins (macrocyclic component) [41,42,43,44]. The addition of Pd and Pt complexes to the mixture of cyclodextrins and ω-(3,5-dimethylphenyl)-4-alkyl-4,4′-bipyridine yielded rotaxanes containing the metal complex of the bipyridinium ligand as the axle component. The rotaxanes and pseudorotaxanes form micelles in aqueous media because of their tendency to aggregate. Our goal is to use the aggregated rotaxanes as a precursor to metal nanoparticles. The reduction of micellar aggregates of transition metal complexes has been reported as an efficient procedure for preparing metal nanoparticles under mild conditions. Lee reported that a mixture of an organic surfactant and H_2_PtCl_6_ was reduced by dodecanol to form Pt nanoparticles with a diameter of 1.7 ± 0.5 nm [45]. The reduction of a mixture of HAuCl_4_ and a cationic surfactant dispersed in water was found to produce Au nanoparticles with a diameter of 1.9 ± 0.3 nm [46]. Chaudhary employed metallomicelles of a Pd(II) complex with dodecylamine ligands as the precursor to Pd nanoparticles, and obtained a product with a diameter of 3.4–4.0 nm [47].

**Scheme 1:**
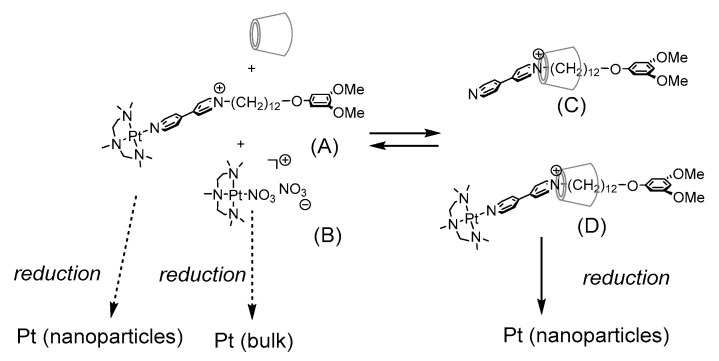
Formation of Pt-containing rotaxanes and Pt nanoparticles in this study.

Scheme 1 outlines the formation of Pt nanoparticles in this work. Mixing cyclodextrin, a Pt(II) complex with *N*-alkylbipyridinium ligand (A), and nitrate-platinum complex (B) in aqueous media forms pseudorotaxane of cyclodextrin and the dissociated ligand (C), as well as Pt-containing rotaxane (D). Both Pt complexes (A) and (D) are aggregated in micellar form in aqueous media, and they are converted into Pt nanoparticles upon reduction. We planned to use several mixtures of (A) and excess amounts of (B) and cyclodextrins as a precursor to Pt nanoparticles. Different amounts of the starting compounds shift the equilibrium to depress the formation of the CD-free complex (A) and pseudorotaxane (C). Thus, the reduction of the mixture would yield Pt nanoparticles from the rotaxane (D) rather than a CD-free complex (A). The reduction of complex (B) also forms Pt, but in bulk, because of the dissolution of the complex in aqueous media. In this paper, we report the conversion of a Pt complex with a rotaxane structure into Pt nanoparticles by NaBH_4_ reduction, as well as the characterization of the rotaxanes in micellar aggregates and metal nanoparticles.

## 2. Results and Discussion

### 2.1. Preparation and Characterization of the Cyclodextrin Rotaxanes of the Pt Complex

**Scheme 2:**
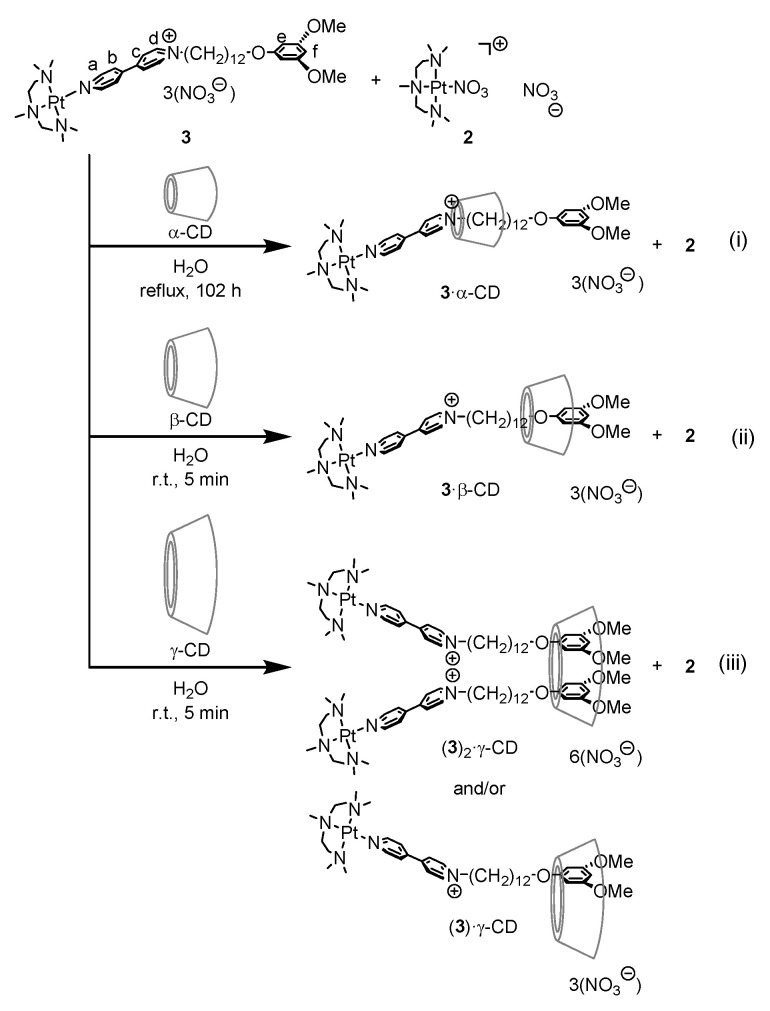
Preparation of cyclodextrin rotaxanes. Symbols a-f in complex **3** denote aromatic hydrogens at the respective positions. They correspond to the NMR spectra in Figure 1(K) and Figure 2(N). (i)–(iii) show the rotaxane or pseudorotaxane obtained from α-, β-, and γ-cyclodextrins, respectively.

A reaction of 4,4′-bipyridinium derivative having a ω-(3,5-methoxyphenoxy)dodecyl substituent at a pyridine nitrogen (NC_6_H_4_-C_6_H_4_N-(CH_2_)_12_-O(C_6_H_3_-3,5-(OMe)_2_)^+^ ([1]^+^) with a Pt complex having a 1,1,4,7,7-pentamethyl-1,4,7-triaza-heptane (Me_5_dien) ligand (Pt(NO_3_)(Me_5_dien))^+^ ([2]^+^) in the presence of AgNO_3_ affords a complex containing the amphiphilic ligand (Pt(Me_5_dien)(**1**)](NO_3_)_3_ ([3](NO_3_)). The addition of α-cyclodextrin to a mixture of [3](NO_3_)_3_ and [2](NO_3_) (molar ratio of 2:1:2), followed by heating the resulting aqueous solution for 102 h, caused the formation of [2]rotaxane ((**3**)(α-CD)](NO_3_)_3_ as shown in Scheme 2(i). ^1^H-nuclear magnetic resonance (NMR) spectrum of the reaction mixture suggested the complete complexation of the bipyridinium ligand to the Pt center and the absence of bare alkylbipyridinium or its organic pseudorotaxane with α-CD ([1]^+^ and [(**1**)(α-CD)]^+^). The reaction without the addition of extra [2] was very slow and did not reach completion even after prolonged heating. An excess amount of [2]^+^ in the mixture enhanced the formation of [(**3**)(α-CD)](NO_3_)_3_ via the reversible dissociation and association of the bipyridinium ligand. Complexes [3]^3+^ and [2]^+^ undergo a mutual exchange of the bipyridinium ligand, and rapidly form a rotaxane via the dissociation of the ligand of [3]^3+^, formation of pseudorotaxane [(**1**)(α-CD)]+, and re-coordination of the pseudorotaxane to the Pt center of [2]^+^.

The individual reaction of β-cyclodextrin and γ-cyclodextrin with a mixture of [3](NO_3_)_3_ and [2](NO_3_) (molar ratio: 2:1:1 for the former and 1:2:4 for the latter) afforded the respective cationic rotaxanes [(**3**)(β-CD)](NO_3_)_3_ and [(**3**)_2_(γ-CD)_2_](NO_3_)_6_ within 5 min (r.t.) (Scheme 2(ii), (iii)). The rapid formation of the rotaxanes is ascribed to the large ring size of β- and γ-CD, which enables slipping synthesis of the rotaxanes. Isolation of the resulting rotaxanes was not successful because of the reversible formation and degradation of the rotaxanes in the solution. We conducted a titration study of the formation of [(**3**)(β-CD)](NO_3_)_3_ and [(**3**)_2_(γ-CD)_2_](NO_3_)_6_ by using ^1^H-NMR spectroscopy in order to obtain further insight of the reaction.

**Figure 1:**
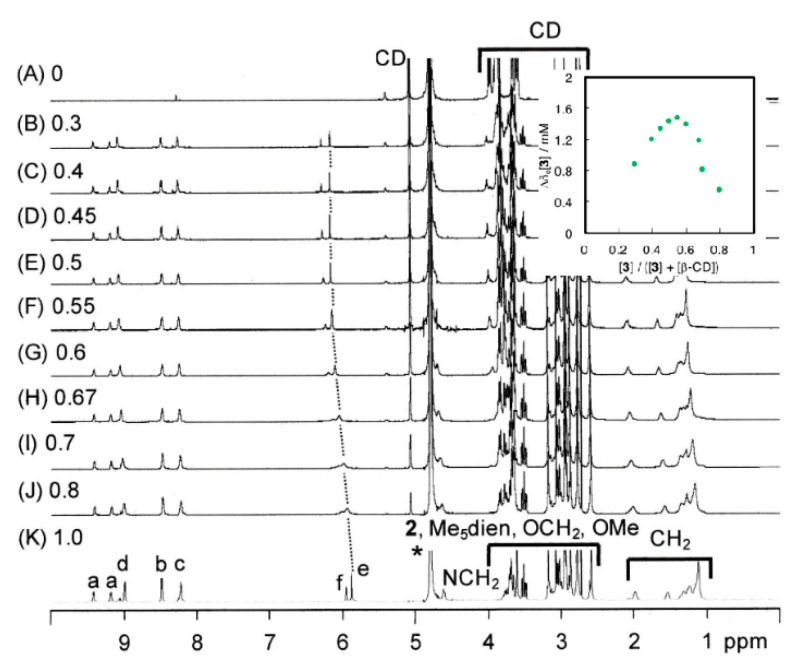
^1^H-nuclear magnetic resonance (NMR) spectra of the mixtures of [3]^3+^ and β-cyclodextrin. (D_2_O, r.t., 500 MHz). [3]/([3] + [β-CD]) = (**A**) 0, (**B**) 0.3, (**C**) 0.4, (**D**) 0.45, (**E**) 0.5, (**F**) 0.55, (**G**) 0.6, (**H**) 0.67, (**I**) 0.7, (**J**) 0.8, (**K**) 1.0. Signals a-f are assigned to the hydrogens of complex **3**, in Scheme 2. A signal with asterisk is due to HDO in the solvent. Job’s plots based on the positions of the signal (e) are shown in the inset.

Figure 1 presents the results of titration using β-cyclodextrin. The aromatic hydrogen signals (e, f) of [3]^3+^ (K) are shifted to lower magnetic field positions upon the increase of β-cyclodextrin concentration (the total concentration of [3]^3+^ and β-CD are 10.0 mM). Job’s plots based on the change of the ^1^H-NMR signal due to aromatic hydrogen e suggest the aggregation of the complex and β-CD close to 1:1 stoichiometry (inset of Figure 1).

**Figure 2:**
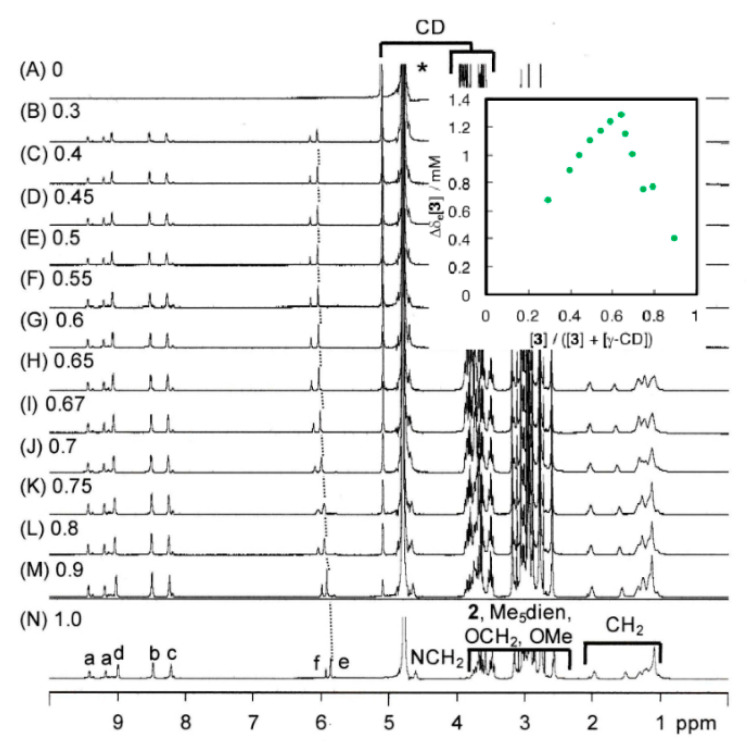
^1^H-NMR spectra of mixtures of [3]^3+^ and γ-cyclodextrin. (D_2_O, r.t., 500 MHz). [3]/([3] + [γ-CD]) = (**A**) 0, (**B**) 0.3, (**C**) 0.4, (**D**) 0.45, (**E**) 0.5, (**F**) 0.55, (**G**) 0.6, (**H**) 0.65, (**I**) 0.67, (**J**) 0.7, (**K**) 0.75, (**L**) 0.8, (**M**) 0.9, (**N**) 1.0. Signals a-f are assigned to the hydrogens of complex **3**, in Scheme 2. A signal with asterisk is due to HDO in the solvent. Job’s plots based on the positions of the signal (e) are shown in the inset.

Complex [3]NO_3_ and γ-cyclodextrin could form not only [3]rotaxane [(**3**)_2_(γ-CD)]^6+^ via a 1:2 aggregation of the macrocyclic and axle components, but also [2]rotaxane [(**3**)(γ-CD)]^3+^ by a 1:1 aggregation in the solution. We conducted titration of the two components in the NMR samples, whose results are shown in Figure 2. The ^1^H NMR signals of complex [3]NO_3_, particularly those of the aromatic hydrogens, shift towards lower magnetic field positions. Job’s plots of the signal positions show a maximum close to 0.65, which is consistent with the 1:2 aggregation (inset of Figure 2). Thus, complex [3]^3+^ forms majorly [3]rotaxane [(**3**)_2_(γ-CD)]^6+^, and a negligible amount of [2]rotaxane [(**3**) (γ-CD)]^3+^ in the solution.

**Figure 3:**
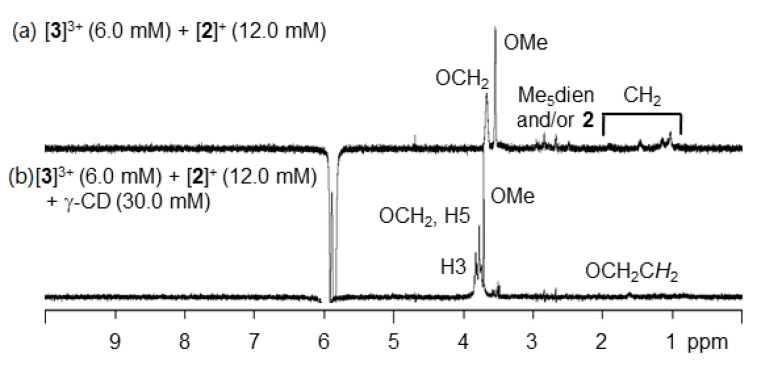
^1^H-NMR spectra with the rotating-frame Overhauser effect (ROE) difference mode. (**a**) [3]^3+^ (6.0 mM) + [2]^+^ (12.0 mM). (**b**) [3]^3+^ (6.0 mM) + [2]^+^ (12.0 mM) + γ-CD (30.0 mM).

The positions of the aromatic hydrogen signals are influenced by the ring size of the cyclodextrins. Figure 3 compares the spectrum of the mixture of [(**3**)_2_(γ-CD)_2_]^6+^ and [2]^+^ with the rotating-frame Overhauser effect (ROE) mode. The irradiation of the aromatic hydrogen signals of the terminal group enhanced the signals at 3.8 ppm, assigned to the hydrogens of γ-CD (at the interior side of the ring). This suggests that the 3,5-dimethoxyphenyl group of the Pt complex is at a proximity to γ-CD.

**Figure 4:**
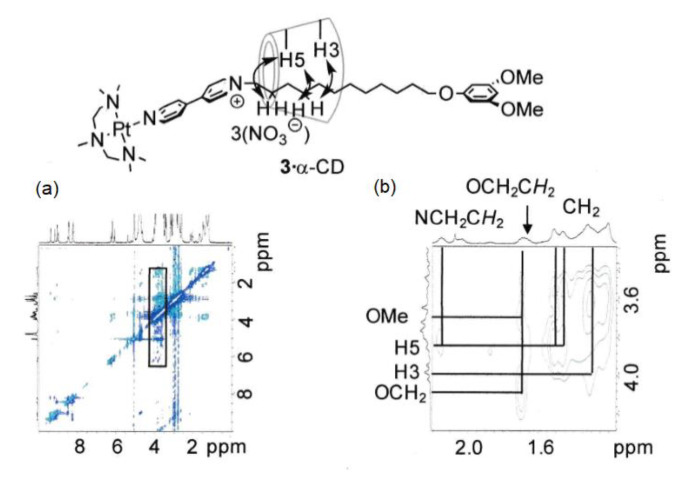
ROESY NMR spectra of [(**3**)·α-CD]^3+^ (D_2_O, 25 °C, 500 MHz). Total spectrum (**a**) and expanded spectrum (**b**).

**Figure 5:**
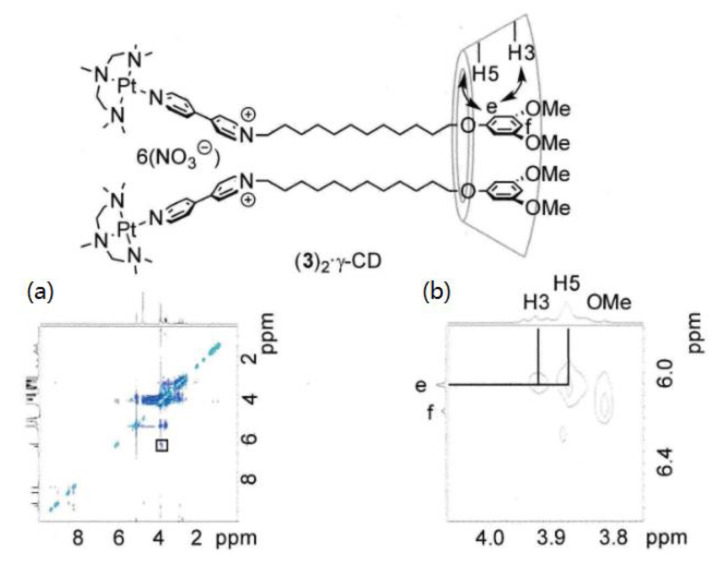
ROESY NMR spectra of [(**3**)_2_·γ-CD]^6+^ (D_2_O, 25 °C, 500 MHz). Total spectrum (**a**) and expanded spectrum (**b**).

More information on the relative positions of the axle molecules and cyclodextrins was obtained by two-dimensional rotating frame Overhauser effect spectroscopy (ROESY) NMR spectroscopy experiments; the results are shown in Figure 4 and Figure 5.

Figure 4(a), (b) present the cross peaks between the signals due to the CH_2_ group hydrogens and the signals assigned to the interior hydrogens of α-CD (3.8 and 4.0 ppm) with high intensity. The NCH_2_ hydrogens show significant correlation with the signals of α-CD. Considerably low-intensity cross-peaks are observed at the positions between the aromatic hydrogen signals of the terminal group of the ligand (e, f) and the interior hydrogens of α-CD. Thus, the rotaxane prefers the structure with the macrocyclic component (α-CD) at proximity to the CH_2_ hydrogens adjacent to the bipyridinium group. Another structure with the terminal 3,5-dimethylphenyl group and α-CD is also observed in a much smaller amount. Figure 5 shows the spectra of a mixture containing the rotaxane composed of the Pt complex and γ-CD. Clear correlation peaks are observed, indicative of the interaction between the cyclodextrin hydrogen and 3,5-dimethoxyphenyl hydrogens (e, f) and no cross-peak between the cyclodextrin and the CH_2_ hydrogens. The ROESY NMR spectra of the rotaxane containing β-cyclodextrin also shows the cross-peak, indicative of the interaction between the aromatic hydrogens of the terminal group and the hydrogens of cyclodextrin.

**Figure 6:**
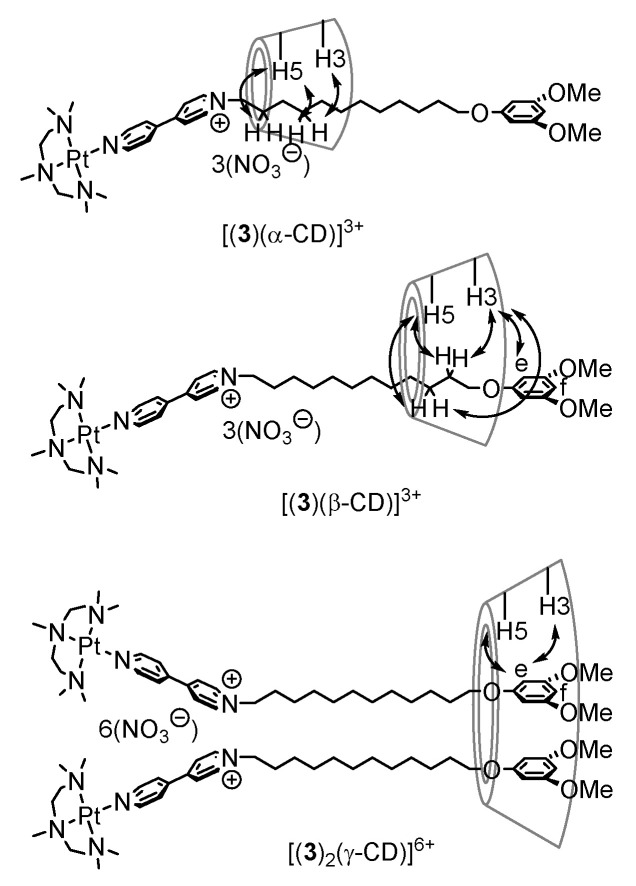
Conformation of polyrotaxanes based on ROESY NMR spectra measurement. Symbols e and f are due to the aromatic hydrogens at the positions. They correspond to the structures in Scheme 2.

Thus, we propose a main conformation of the rotaxanes with α-, β-, and γ-cyclodextrins, as shown in Figure 6. [(**3**)(α-CD)]^3+^ contains the rotaxane having interaction between the CH_2_ groups adjacent to the bipyridinium group of the ligand and α-cyclodextrin in the main, although a minor structure with the terminal 3,5-dimethoxyphenyl group and α-CD is also formed. The rotaxanes with β-, and γ-cyclodextrins have structures with an attractive interaction between the terminal aromatic group and cyclodextrins.

**Figure 7:**
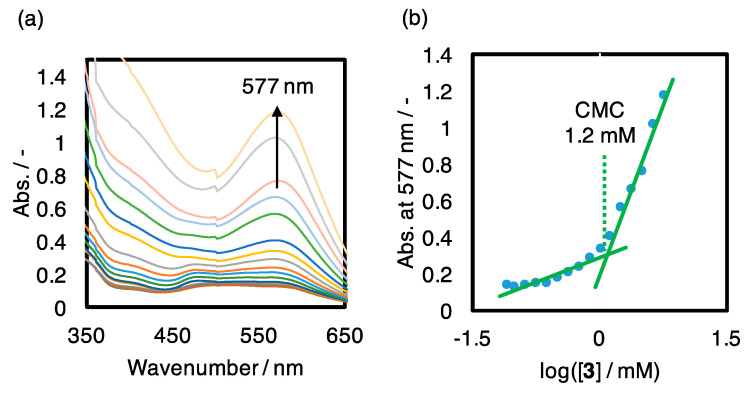
(**a**) Absorption spectra of nile red depending on coexisting complex **3** in aqueous solutions ([3] = 0.08–5.68 mM, [nile red] = 10 µM). (**b**) Plots of absorbance at 577 nm vs. the concentration of **3**.

Complex [3]^3+^ with the cationic metal center and a long alkylene group in the ligand is expected to show an amphiphilic nature and to be aggregated in aqueous solution. The absorption spectra of the complex in aqueous solutions containing nile red showed absorption at 577 nm. Plots of the absorbance depending on the concentration of complex **3** (Figure 7a) shows an inflection point at [3] = 1.2 mM, as shown in Figure 7b. It suggests formation of micelles above the concentration, and solubilization of the dye molecules in the micelles above the critical micelle concentration.

We prepared the samples for transmission electron microscopy (TEM) and atomic force microscopy (AFM) measurements by dropping the solution on carbon-coated Cu grids and removing the water at room temperature.

**Figure 8:**
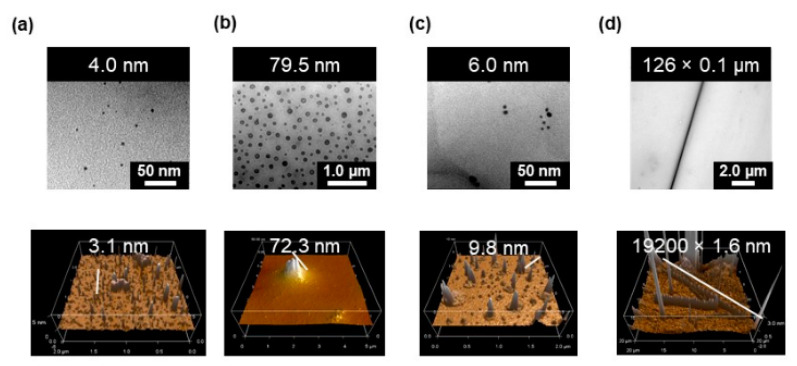
Transmission electron microscopy (TEM) images (upper) and AFM image (lower) for each sample. Measurement was conducted after dropping the solution on the surface of the carbon-coated Cu grids. (**a**) [3]^3+^ = 6.0 mM, [2]^+^ = 12.0 mM. (**b**) [(**3**)·α-CD]^3+^ = 3.4 mM, [3]^3+^ = 2.6 mM, [2]^+^ = 12.0 mM, [α-CD] = 8.6 mM. (**c**) [(**3**)·β-CD]^3+^ = 6.0 mM, [2]^+^ = 12.0 mM. (**d**) [(**3**)_2_·γ-CD]^6+^ = 3.0 mM, [2] = 12.0 mM.

Figure 8 shows the TEM and AFM images obtained from the solutions of [3](NO_3_), [(**3**)(α-CD)](NO_3_)_3_, [(**3**)(β-CD)](NO_3_)_3_, and [(**3**)_2_(γ-CD)](NO_3_)_6_ in aqueous media ([Pt] = 6.0 mM). The spots in the TEM images correspond to the Pt atoms in the complexes and rotaxanes. The average diameters of the spots are estimated as follows: [3](NO_3_) = 4.0 ± 1.1 nm, [(**3**)(α-CD)](NO_3_)_3_ = 79.5 ± 15.1 nm, and [[3](β-CD)](NO_3_)_3_ = 6.0 ± 1.3 nm. The rotaxane [(**3**)_2_(γ-CD)](NO_3_)_6_ provided elongated spots with a size of 125,800 × 108 nm, which were assigned to the rod micelles formed in the solution. The spots observed in the TEM image of [(**3**)(α-CD)](NO_3_)_3_ are much larger than the aggregates of [3](NO_3_) and [(**3**)(β-CD)](NO_3_)_3_. Many of the spots show contrast between a dark particle edge and less-dark central region. This suggests the formation of vesicles by aggregated rotaxanes. Since the solutions contain both rotaxanes and complex [2]^+^ added to enhance the formation of the rotaxane, the aggregates, observed in the TEM images, also contain the rotaxane and the cationic complex without bipyridinium ligands. The size and shape of the aggregates observed by the micrograph differ depending on the size of CDs, in spite of the co-existence of complex [2]^+^. The formed aggregates have a regulated size and different sizes and shapes depending on the CD involved in the rotaxane molecules, despite the presence of such impurities.

### 2.2. Preparation of Pt Nanoparticles

The addition of NaBH_4_ to a solution of the Pt complex with and without CDs afforded Pt nanoparticles via reduction of the Pt(II) compound. The original aqueous solution was yellow; upon the addition of NaBH_4_ (30 equiv./Pt) dispersed in H_2_O (1.0 mL), the color changed to blue at first, and subsequently to brown. The precipitation of a solid material was not observed. The resulting aqueous solution was dropped onto the Cu grid and was analyzed by TEM after removing the water.

**Figure 9:**
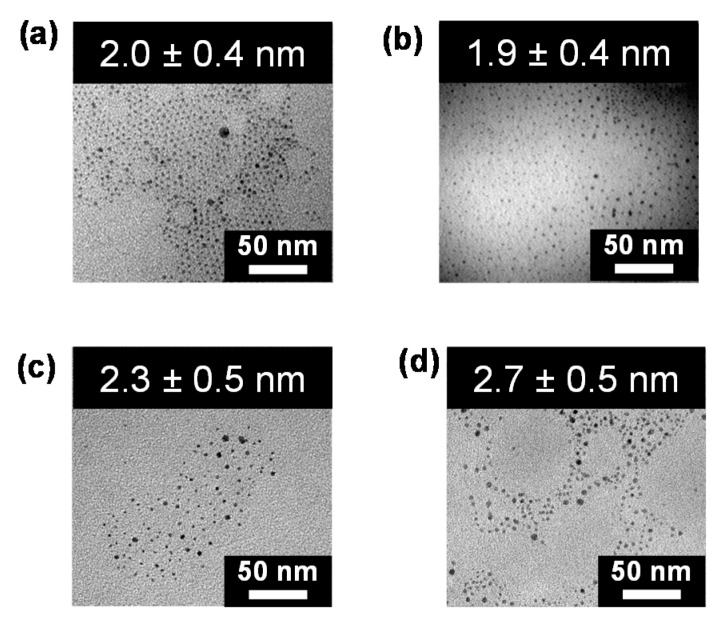
TEM images of the Pt nanoparticles obtained from the reduction of the complex or rotaxane in an aqueous solution (3.0 mM). Measurement was conducted after dropping the solution on the surface of carbon-coated Cu grids. (**a**) [3](NO_3_), (**b**) [[3](α-CD)](NO_3_)_3_, (**c**) [[3](β-CD)](NO_3_)_3_, (**d**) [[3]_2_(γ-CD)](NO_3_)_6_.

Figure 9 presents the TEM images of the Pt particles obtained by the reduction of [3]^3+^ and its rotaxanes with α, β, γ-CDs at 3.0 mM Pt. In all cases, the measurements revealed the presence of sphere-shaped spots with similar sizes. The aggregation of the Pt complex and its rotaxanes, as micelles, vesicles, and rod-micelles, is not directly related to the shape of the Pt nanoparticles obtained by the reduction. The average radii of the nanoparticles obtained from [3]^3+^ increase slightly with an increase in the concentration, 1.4 ± 0.4 nm (0.05 mM), 1.8 ± 0.5 nm (0.75 mM), 1.9 ± 0.4 nm (1.5 mM), 2.0 ± 0.4 nm (3.0 mM), 3.1 ± 0.8 nm (6.0 mM). The average size of the formed Pt particles are as follows: 1.3 ± 0.4 nm (0.05 mM), 1.8 ± 0.5 nm (0.75 mM), 1.9 ± 0.4 nm (1.5 mM), 2.0 ± 0.4 nm (3.0 mM), 2.3 ± 0.8 nm (6.0 mM) for [(**3**)(α-CD)](NO_3_)_3_, 1.7 ± 0.4 nm (0.05 mM), 2.0 ± 0.5 nm (0.75 mM), 2.1 ± 0.3 nm (1.5 mM), 2.6 ± 0.4 nm (3.0 mM), and 2.9 ± 0.5 nm (6.0 mM) for [[3](β-CD)](NO_3_)_3_, and 1.9 ± 0.4 nm (0.05 mM), 2.2 ± 0.5 nm (0.75 mM), 2.5 ± 0.3 nm (1.5 mM), 2.7 ± 0.5 nm (3.0 mM), and 2.8 ± 0.5 nm (6.0 mM) for [(**3**)_2_(γ-CD)](NO_3_)_3_. The treatment of a mixture of ligand ([1]^+^, 6.0 mM), Pt complex ([2]^+^, 18 mM), and α-CD (12 mM) in an aqueous solution with NaBH_4_ did not form the nanoparticles, but caused the deposition of a black solid composed of metallic Pt.

**Figure 10:**
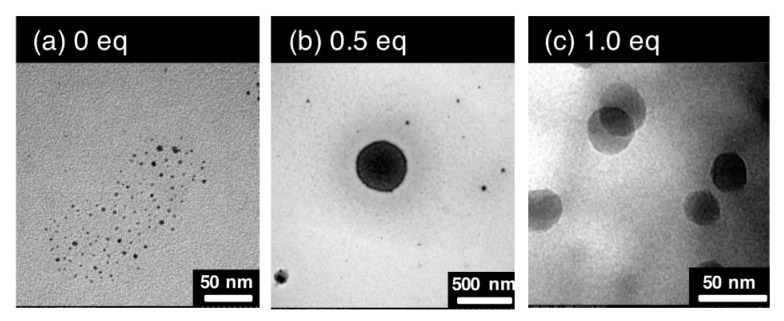
TEM images of the Pt nanoparticles obtained by NaBH_4_ reduction of [(**3**)(β-CD)](NO_3_)_3_ in an aqueous solution (3.0 mM): (**a**) as prepared, (**b**) after addition of tetrakis(4-carboxyphenyl)porphyrin (TCPP) (0.5 eq to Pt), (**c**) after further addition of TCPP (1.0 eq to Pt).

The reduction of [(**3**)(β-CD)](NO_3_)_3_ by NaBH_4_ in the presence of tetrakis(4-carboxyphenyl)porphyrin (TCPP) formed Pt particles with a much larger particle size than those without addition of TCPP, as shown in the TEM images (Figure 10a–c). TCPP and the Pt complex compete for coordination with β-CD because both can form rotaxane or pseudorotaxane with β-CD. The high stability of the Pt nanoparticles from the rotaxanes in this study is partly attributed to the protection of the cyclodextrins on the surface of the Pt particles. Stable metal nanoparticles protected by functionalized cyclodextrins were synthesized and used as efficient catalysts for synthetic organic reactions [48,49,50].

Thermal stability of Pt nanoparticles obtained by the above procedures was compared. The particles obtained from the reduction of complex [3]^+^ are stable at room temperature; however, their particle size increases on heating the solution at 70 °C for 3 h (from 2.0 ± 0.4 nm to 3.2 ± 0.6 nm). The size of the Pt particles obtained from the rotaxanes, however, did not change even after heat treatments: 1.9 ± 0.4 nm to 2.0 ± 0.4 nm for [(**3**)(α-CD)](NO_3_)_3_, 2.6 ± 0.4 nm to 2.5 ± 0.5 nm for [(**3**)(β-CD)](NO_3_)_3_, and 2.8 ± 0.6 nm to 2.8 ± 0.6 nm for [(**3**)_2_(γ-CD)](NO_3_)_3_.

## 3. Materials and Methods

### 3.1. General

All the chemicals were commercially available from Tokyo Kasei Chemicals Co. ^1^H- and ^13^C{^1^H}-NMR spectra were acquired on a Bruker AV-400M (400 MHz) and a JEOL JNM-500 (500 MHz). The chemical shifts were referenced with respect to CHCl_3_ (δ 7.26), HDO (δ 4.79) for ^1^H, and CDCl_3_ (δ 77.0), DSS (sodium 3-(trimethylsilyl)-1-propanesulfonate) (δ 0.0) for ^13^C as internal standards. High resolution electrospray ionization mass spectrometry (HR-ESI-MS) was recorded on a Bruker micrOTOF II. The TEM was performed at 100 kV using Hitachi H-7650 Zero with collodion/carbon-support film grids, COL-C15 STEM Cu150P (Okenshoji, Japan). The AFM was performed using Oxford Instruments Cypher S using OLYMPUS AC160TS.

### 3.2. Preparation of [4,4′-bpy-N-(CH_2_)_12_OC_6_H_3_-3,5-(OMe)_2_][Br] ([1]^+^Br^−^) and [4,4′-bpy-N-(CH_2_)_12_OC_6_H_3_-3,5-(OMe)_2_][NO_3_] ([1]^+^(NO_3_) ^−^)

The title compound with Br^−^ was prepared according to the literature. ^1^H-NMR (400 MHz, D_2_O, r.t.): δ 0.88–1.26 (m, 16H, CH_2_), 1.39 (quin, 2H, OCH_2_C*H*_2_, *J* = 6.6 Hz), 1.95 (quin, 2H, NCH_2_C*H*_2_, *J* = 6.6 Hz), 3.54 (t, 2H, OCH_2_, *J* = 7.0 Hz), 3.56 (s, 6H, OCH_3_), 4.67 (t, 2H, NCH_2_, *J* = 6.8 Hz), 5.73 (s, 2H, C_6_H_3_), 5.90 (s, 1H, C_6_H_3_), 7.83 (d, 2H, C_10_H_8_N_2_, *J* = 6.3 Hz), 8.36 (d, 2H, C_10_H_8_N_2_, *J* = 6.6 Hz), 8.75 (d, 2H, C_10_H_8_N_2_, *J* = 6.2 Hz), 8.94 (d, 2H, C_10_H_8_N_2_, *J* = 6.8 Hz). ^13^C{^1^H}-NMR (125 MHz, CDCl_3_, r.t.): δ 26.1 (CH_2_), 26.3 (CH_2_), 29.2 (CH_2_), 29.3 (CH_2_), 29.4 (CH_2_), 29.5 (CH_2_), 29.6 (CH_2_), 29.7 (CH_2_), 29.9 (CH_2_), 30.0 (CH_2_), 55.5 (OCH_2_), 62.1 (NCH_2_), 68.2 (OCH_3_), 92.9 (C_6_H_3_), 93.5 (C_6_H_3_), 121.6 (C_10_H_8_N_2_), 125.9 (C_10_H_8_N_2_), 141.1 (C_10_H_8_N_2_), 145.8 (C_10_H_8_N_2_), 151.7 (C_10_H_8_N_2_), 154.0 (C_10_H_8_N_2_), 161.2 (C_6_H_3_), 161.6 (C_6_H_3_).

Compound with NO_3_ anion. ^1^H-NMR (500 MHz, D_2_O, r.t.): δ 0.84–1.23 (m, 16H, CH_2_), 1.34 (quin, 2H, OCH_2_C*H*_2_, *J* = 6.4 Hz), 1.95 (quin, 2H, NCH_2_C*H*_2_, *J* = 6.9 Hz), 3.49 (s, 6H, OCH_3_), 3.49 (brs, 2H, OCH_2_), 4.70 (t, 2H, NCH_2_, *J* = 7.2 Hz), 5.68 (s, 2H, C_6_H_3_), 5.81 (s, 1H, C_6_H_3_), 7.84 (d, 2H, C_10_H_8_N_2_, *J* = 5.5 Hz), 8.38 (d, 2H, C_10_H_8_N_2_, *J* = 6.5 Hz), 8.72 (d, 2H, C_10_H_8_N_2_, *J* = 5.0 Hz), 9.00 (d, 2H, C_10_H_8_N_2_, *J* = 6.5 Hz). ^1^H-NMR (400 MHz, CDCl_3_, r.t.): δ 1.17–1.48 (m, 16H, CH_2_), 1.72 (brs, 2H, OCH_2_C*H*_2_), 2.01 (brs, 2H, NCH_2_C*H*_2_), 3.74 (s, 6H, OCH_3_), 3.88 (t, 2H, OCH_2_, *J* = 7.9 Hz), 4.76 (t, 2H, NCH_2_, *J* = 6.2 Hz), 6.05 (s, 2H, C_6_H_3_), 6.06 (s, 1H, C_6_H_3_), 7.67 (d, 2H, C_10_H_8_N_2_, *J* = 4.6 Hz), 8.28 (d, 2H, C_10_H_8_N_2_, *J* = 5.5 Hz), 8.85 (brs, 2H, C_10_H_8_N_2_, *J* = 5.0 Hz), 9.19 (d, 2H, C_10_H_8_N_2_, *J* = 5.8 Hz).

### 3.3. Preparation of [Pt(Me_5_dien)Cl]Cl ([2]Cl)

The complex was prepared by slight modification of the literature method. MeOH (30 mL) was added to Me_5_dien (2.0 mL, 9.5 mmol) to form a solution. Further addition of *cis*-PtCl_2_(dmso)_2_ (4.0 g, 9.5 mmol), and addition of MeOH (20 mL) was followed by heating the mixture for 6 h under reflux. Cooling the solution at room temperature and evaporation of the solvent left a pale brown solid. Recrystallization of the solid product by acetone/CH_2_Cl_2_ yielded off-white crystals of [Pt(Me_5_dien)Cl]Cl ([2]Cl) (4.1 g, 9.3 mmol, 98%). ^1^H-NMR (500 MHz, D_2_O, r.t.): δ 2.79 (s, 6H, CH_3_, *J*_Pt-H_ = 10.1 Hz), 2.93 (t, 2H, CH_2_, *J* = 3.2 Hz), 2.96 (t, 2H, CH_2_, *J* = 3.7 Hz), 2.98 (s, 6H, CH_3_, *J*_Pt-H_ = 12.5 Hz), 3.11 (s, 3H, CH_3_, *J*_Pt-H_ = 21.0 Hz), 3.50 (td, 2H, CH_2_, *J* = 14.1, 3.5 Hz), 3.72 (td, 2H, CH_2_, *J* = 13.3, 3.9 Hz). ^13^C{^1^H}-NMR (125 MHz, D_2_O, r.t.): δ 48.4 (CH_3_), 54.4 (CH_3_), 55.7 (CH_3_), 64.9 (CH_2_), 70.7 (CH_2_).

Treatment of the product by AgNO_3_ yielded [Pt(Me_5_dien)NO_3_]NO_3_ ([2](NO_3_)) in high yield (168.0 mg, 0.33 mmol, 85%). ^1^H-NMR (400 MHz, D_2_O, r.t.): δ 2.79 (s, 6H, CH_3_, *J*_Pt-H_ = 10.3 Hz), 2.94 (t, 2H, CH_2_, *J* = 3.8 Hz), 2.96 (s, 6H, CH_3_), 2.97 (t, 2H, CH_2_, *J* = 3.5 Hz), 3.09 (s, 3H, CH_3_, *J*_Pt-H_ = 22.5 Hz), 3.52 (td, 2H, CH_2_, *J* = 14.0, 3.8 Hz), 3.70 (td, 2H, CH_2_, *J* = 13.3, 4.2 Hz). ^13^C{^1^H}-NMR (125 MHz, D_2_O, r.t.): δ 48.4 (CH_3_), 54.4 (CH_3_), 55.7 (CH_3_), 64.9 (CH_2_), 70.7 (CH_2_).

### 3.4. Preparation of [Pt(Me_5_dien)(4,4′-bpy-N-(CH_2_)_12_OC_6_H_3_-3,5-(OMe)_2_)][NO_3_]_3_ ([3](NO_3_))

A suspension of [1](NO_3_) (372 mg, 0.67 mmol) in water (100 mL) was stirred, and [2]Cl (879 mg, 2.0 mmol) and AgNO_3_ (792 mg, 4.67 mmol) were added to the mixture, and mixed with water (50 mL). Water (50 mL) was added to the mixture, and the resulting suspension was heated for 66 h at 80 °C. After cooling to room temperature, the reaction mixture was filtered to remove AgCl. Evaporation of the solvent from the filtrate produced a brown solid (1.40 g). ^1^H- and ^13^C{^1^H}-NMR spectra of the solid showed that it contained [Pt(Me_5_dien)(4,4′-bpy-*N*-(CH_2_)_12_OC_6_H_3_-3,5-(OMe)_2_)][NO_3_]_3_ (**3**) and **2** in 1:2 molar ratio. ^1^H-NMR (500 MHz, D_2_O, r.t.): δ 1.00–1.40 (m, 16H, CH_2_), 1.57 (quin, 2H, OCH_2_C*H*_2_, *J* = 6.7 Hz), 2.01 (quin, 2H, NCH_2_C*H*_2_, *J* = 6.5 Hz), 2.50–3.20 (19H, NCH_3_, NCH_2_), 3.43–3.80 (4H, NCH_2_), 3.54 (s, 6H, OCH_3_), 3.73 (brs, 2H, OCH_2_), 4.64 (t, 2H, NCH_2_, *J* = 7.2 Hz), 5.96 (s, 2H, C_6_H_3_), 6.02 (s, 1H, C_6_H_3_), 8.22 (d, 2H, C_10_H_8_N_2_, 4.5), 8.47 (d, 2H, C_10_H_8_N_2_, *J* = 7.0 Hz), 9.01 (d, 2H, C_10_H_8_N_2_, *J* = 6.5 Hz), 9.17 (d, 1H, C_10_H_8_N_2_, *J* = 5.5 Hz), 9.41 (d, 1H, C_10_H_8_N_2_, *J* = 6.0 Hz). ^13^C{^1^H}-NMR (100 MHz, D_2_O, r.t.): δ 30.5 (CH_2_), 31.3 (CH_2_), 31.3 (CH_2_),31.6 (OCH_2_*C*H_2_), 31.7 (CH_2_), 31.8 (CH_2_), 31.8 (2CH_2_) 33.2 (NCH_2_*C*H_2_), 47.6 (NCH_2_), 53.4 (NCH_3_), 55.2 (NCH_3_), 63.9 (NCH_2_), 64.0 (NCH_2_), 69.9 (NCH_2_), 70.3 (OCH_2_), 94.7 (C_6_H_3_), 95.6 (C_6_H_3_), 128.4 (C_10_H_8_N_2_), 128.6 (C_10_H_8_N_2_), 147.3 (C_10_H_8_N_2_), 147.9 (C_10_H_8_N_2_), 153.2 (C_10_H_8_N_2_), 155.6 (C_10_H_8_N_2_), 156.1 (C_10_H_8_N_2_), 163.0 (C_6_H_3_), 163.5 (C_6_H_3_). ESI-MS (eluent:H_2_O): calcd for [C_39_H_64_N_5_O_3_Pt]^2+^ 422.73, found 422.73 (M^2+^).

### 3.5. Rotaxane of α-CD with [Pt(Me_5_dien)(4,4′-bpy-N-(CH_2_)_12_OC_6_H_3_-3,5-(OMe)_2_)][NO_3_]_3_)

A mixture of [3]^+^ and [2]^+^ in 1:2 molar ratio (266.2 mg, **3**: 0.13 mmol, **2**: 0.26 mmol) was dissolved in water (12.0 mL). To the solution was added α-CD (256.8 mg, 0.26 mmol) and water (10.0 mL). The solution was heated for 102 h under reflux. The solution was cooled to room temperature, and the resulting solution was analyzed by NMR spectroscopy and TEM. NMR data were obtained from the equilibrated mixture of the rotaxane and starting materials. ^1^H-NMR (400 MHz, D_2_O, r.t.): δ 1.11–1.77 (m, 16H, CH_2_), 1.70 (quin, 2H, OCH_2_C*H*_2_, *J* = 5.2 Hz), 2.16 (quin, 2H, NCH_2_C*H*_2_, *J* = 6.1 Hz), 2.58–3.14 (19H, NCH_3_, NCH_2_), 3.46–3.80 (4H, NCH_2_), 3.56–3.74 (m, 12H, C_36_H_72_O_36_-H4, -H2), 3.74–3.96 (m, 12H, C_36_H_72_O_36_-H6, -H5), 3.82 (s, 6H, OCH_3_), 3.96–4.07 (m, 6H, C_36_H_72_O_36_-H3), 4.04 (brs, 2H, OCH_2_), 4.75 (t, 2H, NCH_2_, *J* = 8.4 Hz), 5.05–5.11 (m, 6H, C_36_H_72_O_36_-H1), 6.23 (s, 2H, C_6_H_3_), 6.30 (s, 1H, C_6_H_3_), 8.26 (d, 2H, C_10_H_8_N_2_, *J* = 5.8 Hz), 8.48 (d, 2H, C_10_H_8_N_2_, *J* = 6.3 Hz), 9.08 (d, 2H, C_10_H_8_N_2_, *J* = 6.4 Hz), 9.19 (d, 1H, C_10_H_8_N_2_, *J* = 6.1 Hz), 9.42 (d, 1H, C_10_H_8_N_2_, *J* = 5.5 Hz). ESI-MS (eluent:H_2_O): calcd for [C_75_H_124_N_5_O_33_Pt]^2+^ 606.26, found 606.26 (M^2+^).

### 3.6. Rotaxane of β-CD with [Pt(Me_5_dien)(4,4′-bpy-N-(CH_2_)_12_OC_6_H_3_-3,5-(OMe)_2_)][NO_3_]_3_)

A mixture of [3]^+^ and [2]^+^ in 1:2 molar ratio (12.1 mg, **3**: 0.006 mmol, **2**: 0.012 mmol) was dissolved in water (12.0 mL). To the solution was added β-CD (6.8 mg, 0.006 mmol) and water (1.0 mL). Stirring the solution for 5 min yielded a yellow solution. The solution was analyzed by NMR spectroscopy and TEM. NMR data were obtained from the equilibrated mixture of the rotaxane and starting materials. ^1^H-NMR (500 MHz, D_2_O, r.t.): δ 1.24–1.49 (m, 16H, CH_2_), 1.69 (m, 2H, OCH_2_C*H*_2_), 2.12 (m, 2H, NCH_2_C*H*_2_), 2.58–3.18 (19H, NCH_3_, NCH_2_), 3.46–3.80 (4H, NCH_2_), 3.59–3.73 (m, 14H, C_42_H_84_O_42_-H4, -H2), 3.73–3.96 (m, 12H, C_42_H_84_O_42_-H6, -H5), 3.83 (s, 6H, OCH_3_), 3.96–3.90 (m, 6H, C_42_H_84_O_42_-H3), 4.01 (brs, 2H, OCH_2_), 4.72 (t, 2H, NCH_2_, *J* = 7.6 Hz), 5.05–5.11 (d, 6H, C_36_H_72_O_36_-H1, *J* = 3.6 Hz), 6.17 (s, 2H, C_6_H_3_), 6.28 (s, 1H, C_6_H_3_), 8.25 (brs, 2H, C_10_H_8_N_2_, *J* = 5.8 Hz), 8.48 (d, 2H, C_10_H_8_N_2_, *J* = 6.0 Hz), 9.08 (d, 2H, C_10_H_8_N_2_, *J* = 5.4 Hz), 9.18 (d, 1H, C_10_H_8_N_2_, *J* = 6.3 Hz), 9.41 (d, 1H, C_10_H_8_N_2_, *J* = 6.1 Hz).

### 3.7. Rotaxane of γ-CD with [Pt(Me_5_dien)(4,4′-bpy-N-(CH_2_)_12_OC_6_H_3_-3,5-(OMe)_2_)][NO_3_]_3_

Preparation was conducted, similar to the rotaxane with β-CD. NMR data were obtained from the equilibrated mixture of the rotaxane and starting materials. ^1^H-NMR (500 MHz, D_2_O, r.t.): δ 1.02-1.39 (m, 16H, CH_2_), 1.63 (m, 2H, OCH_2_C*H*_2_), 2.04 (m, 2H, NCH_2_C*H*_2_, *J* = 7.4 Hz), 2.58–3.22 (m, 19H, NCH_3_, NCH_2_), 3.59–3.70 (m, 14H, C_48_H_96_O_48_-H4, -H2), 3.66 (td, 2H, NCH_2_, *J* = 13.6, 4.1 Hz), 3.70–3.77 (m, 12H, C_48_H_96_O_48_-H6, -H5), 3.86 (td, 2H, NCH_2_, *J* = 12.7, 3.0 Hz), 3.81 (s, 6H, OCH_3_), 3.77–3.84 (m, 6H, C_48_H_96_O_48_-H3), 3.89 (brs, 2H, OCH_2_), 4.67 (t, 2H, NCH_2_, *J* = 8.3 Hz), 5.09 (d, 6H, C_48_H_96_O_48_-H1, *J* = 3.7 Hz), 5.97 (s, 2H, C_6_H_3_), 5.06 (s, 1H, C_6_H_3_), 8.24 (brs, 2H, C_10_H_8_N_2_), 8.49 (d, 2H, C_10_H_8_N_2_, *J* = 6.6 Hz), 9.04 (d, 2H, C_10_H_8_N_2_, *J* = 6.6 Hz), 9.18 (d, 1H, C_10_H_8_N_2_, *J* = 6.4 Hz), 9.42 (d, 1H, C_10_H_8_N_2_, *J* = 6.2 Hz).

### 3.8. Reduction of Pt Complex and Its Rotaxanes

A solution of [3](NO_3_) (0.1–12 µmol) in water ([3] = 0.1–12.0 mM, 1.0 mL) was prepared. It was treated with a solution of NaBH_4_ (30 eq. to [3], 0.1–13.6 mg, 3.0–360 µmol) in water (3.0–360 mM, 1.0 mL). Stirring the mixture for 17 h at room temperature caused formation of a colorless to pale brown solution. A drop of the solution was transferred to the surface of carbon-coated Cu grids, and the resulting product was analyzed by TEM. Reduction of the rotaxanes to Pt nanoparticles was carried out analogously.

## 4. Conclusions

The Pt complex with a bipyridinium ligand having a long *N*-alkyl substituent forms rotaxanes with α-, β-, and γ-cyclodextrins. These rotaxane aggregate in aqueous media to form micelles or vesicles, depending on the size of the cyclodextrins. The reduction of the Pt(II) complex and its rotaxanes by NaBH_4_ afforded Pt nanoparticles with regulated and small sizes (diameters of 1.3–2.8 nm). The Pt nanoparticles from the cyclodextrins are stable at 70 °C, while those without CDs tended to aggregate in the solution. The protection of the resulting Pt particles by cyclodextrins and/or alkylbipyridnium functioned effectively to stabilize the nanoparticles.
